# Supplementary material for: Short-Term Caloric Restriction and Subsequent Re-Feeding Compromise Liver Health and Associated Lipid Mediator Signaling in Aged Mice
Source: Nutrients. 2023 Aug 21;15(16):3660. doi: 10.3390/nu15163660 (PMC10458887; doi:10.3390/nu15163660)
Supplement: Supplementary file 1 [file nutrients-15-03660-s001.zip › nutrients-2521443_revised_supplement_clean.pdf]

Supplement: Short-term caloric restriction and subsequent re-feeding compromise liver health and associated lipid mediator signaling in aged mice

Patrick Schädel[1#], Mareike Wichmann-Costaganna[1#], Anna Czapka[1,2], Nadja Gebert[3], Alessandro Ori[3], and Oliver Werz[1]

[1] Department of Pharmaceutical/Medicinal Chemistry, Institute of Pharmacy, Friedrich Schiller University Jena, D-07743, Germany

[2] Leibniz Institute for Natural Product Research and Infection Biology, Hans Knöll Institute, Jena, D-07745, Germany

[3] Leibniz Institute on Aging - Fritz Lipmann Institute, Jena, D-07745, Germany

[#] these authors contributed equally to this work.

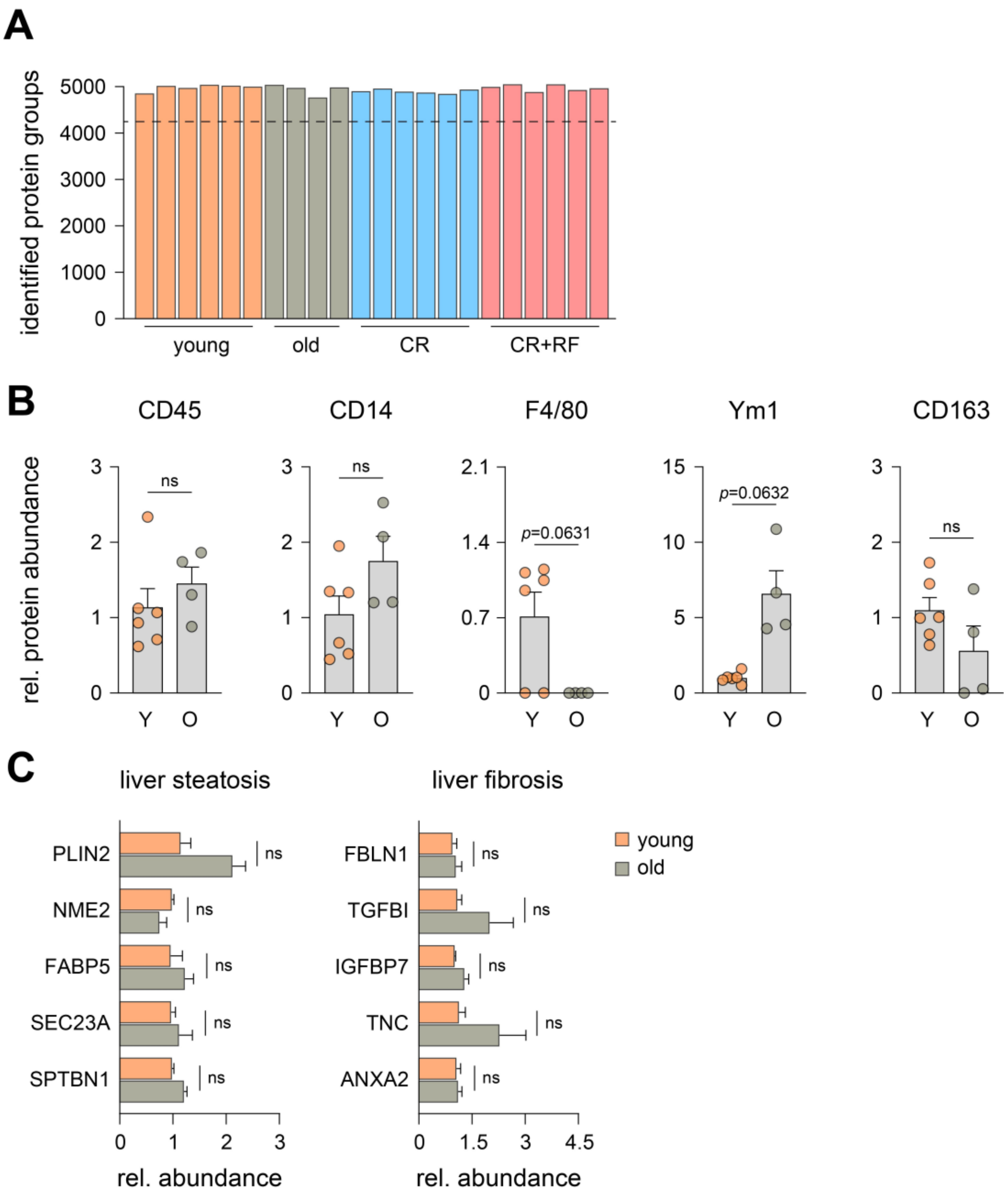

Figure S1: Proteomic profiling of liver samples from young and old mice.

(A) Number of identified protein groups for young (Y), old (O), caloric restricted (CR), and re-fed (CR/RF) mice determined by DIA mass spectrometry (*n*=4-6). Dotted line represents the number of grouped proteins that could be detected in all experimental cohorts. **(B)** Protein levels of CD45, CD14, F4/80, Ym1, and CD163 in the hepatic proteome of young and old mice (Table S1, Y: *n*=6; O: *n*=4). **(C)** Relative protein abundance of biomarkers for liver steatosis and fibrosis [1] in the hepatic proteome of young and old mice (Table S1, Y: *n*=6; O: *n*=4).

Statistics: Data are shown as **(B, C)** mean ± SEM; *p*-values were calculated by one-way ANOVA for multiple comparisons with Tukey's post-hoc test or Brown-Forsythe and Welch ANOVA with Dunnett's T3 post-hoc test (Table S3); ns = not significant,

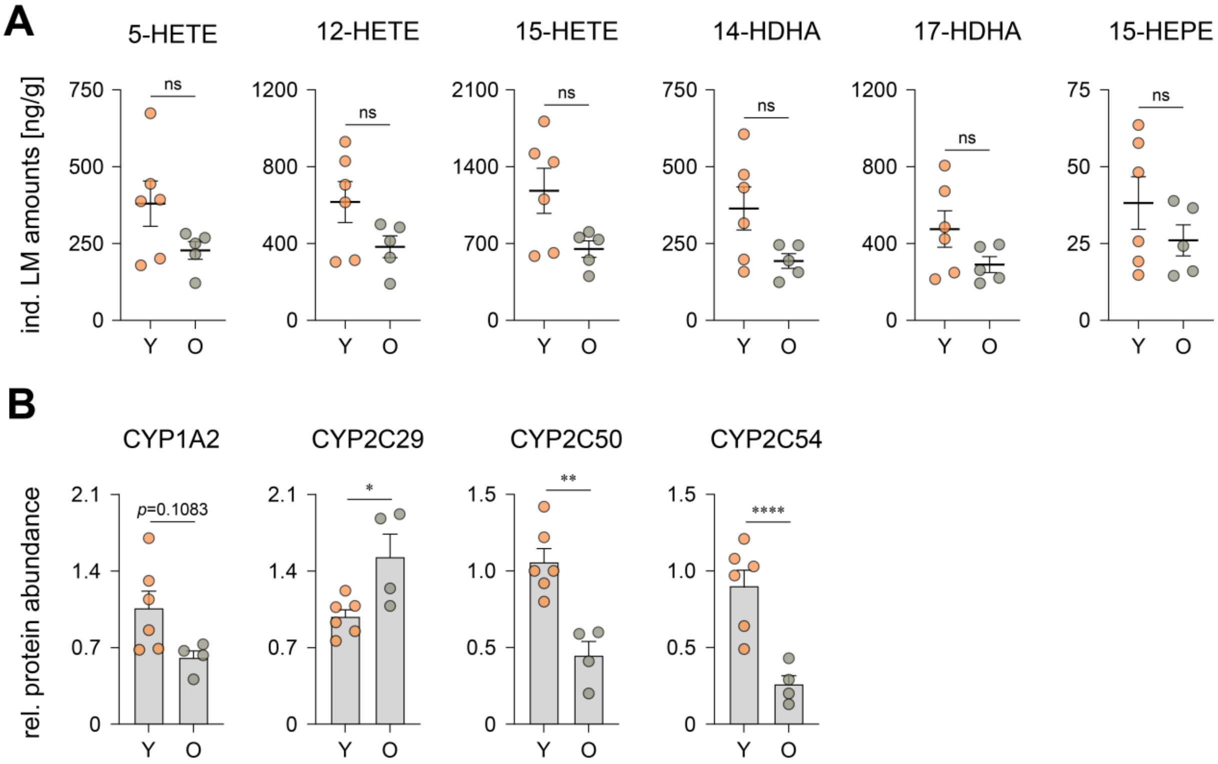

Figure S2: Profiling of PUFA oxygenation in the liver of young and old mice.

(A) Absolute levels of monohydroxylated LM precursors in the liver of young and old mice (Table S2, *n*=6). **(B)** Protein levels of cytochrome P450 isoenzymes in the hepatic proteome of young and old mice (Table S1, Y: *n*=6; O: n=4).

Statistics: Data are shown as **(A, B)** mean ± SEM; p-values were calculated by one-way ANOVA for multiple comparisons with Tukey's post-hoc test or Brown-Forsythe and Welch ANOVA with Dunnett's T3 post-hoc test (Table S3). *$p \leq 0.05$, **$p \leq 0.01$, ****$p \leq 0.0001$, ns = not significant.

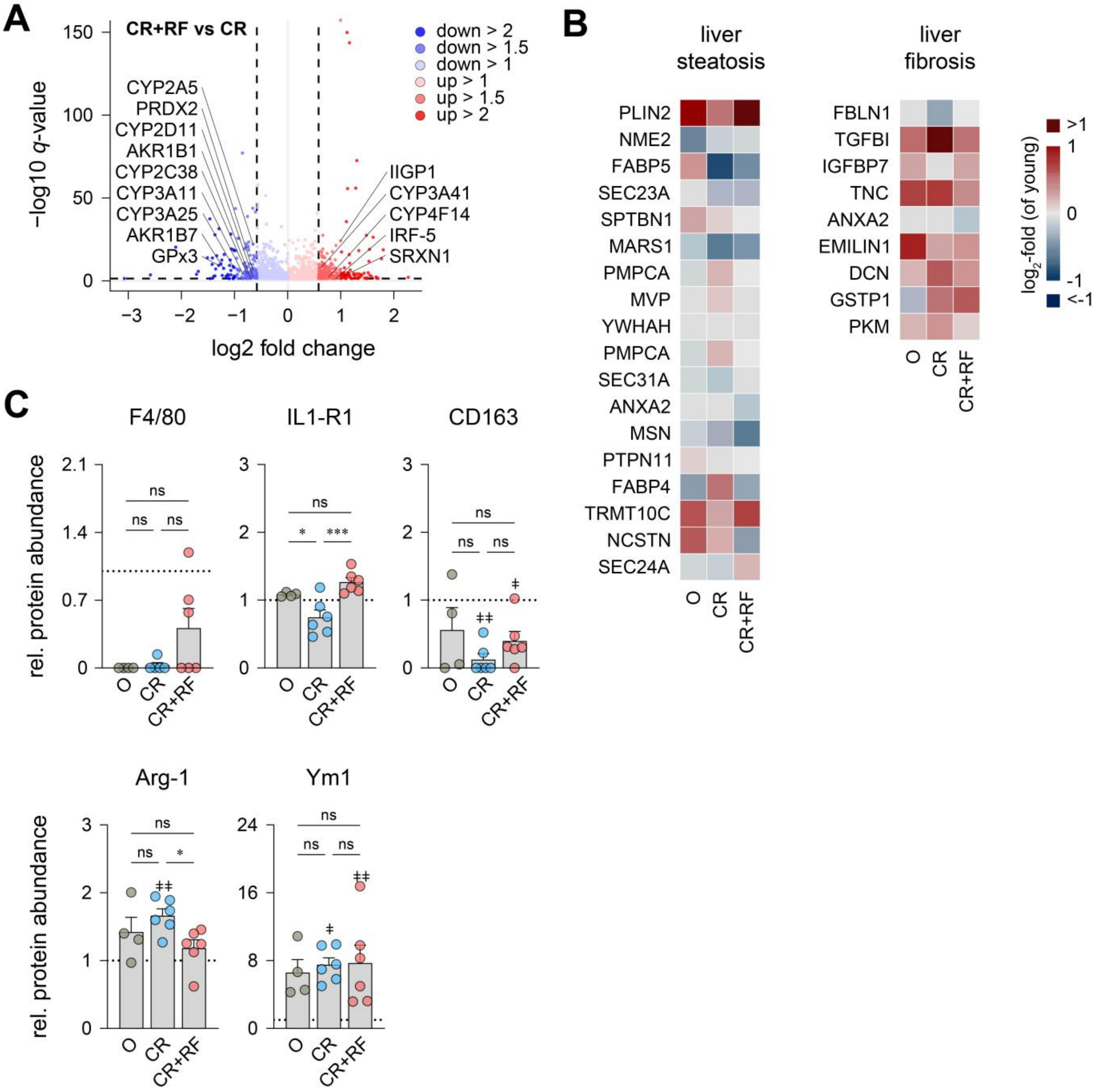

Figure S3: Proteomic profiling of the hepatic inflammatory microenvironment.

(A) Volcano plot of changes to the hepatic proteome in old re-fed (CR+RF) mice in comparison to old caloric restricted (CR) animals (*n*=5-6). Dashed lines indicate cut-offs for significance ($q<0.05$) and absolute fold change (log2>0.58). **(B)** Relative protein abundance of biomarkers for liver steatosis and fibrosis [1] in the hepatic proteome of old, CR and CR+RF mice. Heatmap represents the log2-fold change compared to the median in young mice (Table S1, O: *n*=4; CR, CR+RF: *n*=6). **(C)** Level of innate immune cell markers in the hepatic proteome of old (O), CR, and CR+RF mice (Table S1, O: *n*=4; CR, CR+RF: *n*=6). Dotted line represents the mean value in young mice (Table S1).

Statistics: Data are shown as **(A, B)** median and **(C)** mean ± SEM; p-values were calculated by one-way ANOVA for multiple comparisons with Tukey's post-hoc test or Brown-Forsythe and Welch ANOVA

with Dunnett's T3 post-hoc test (Table S3). Statistical significance is indicated by asterisks for comparisons between CR and O, by hashes for comparisons between CR+RF and O and by ǂ for comparisons with the levels of young mice. *$p \leq 0.05$, **$p \leq 0 .01$, ***$p \leq 0.001$, ns = not significant.

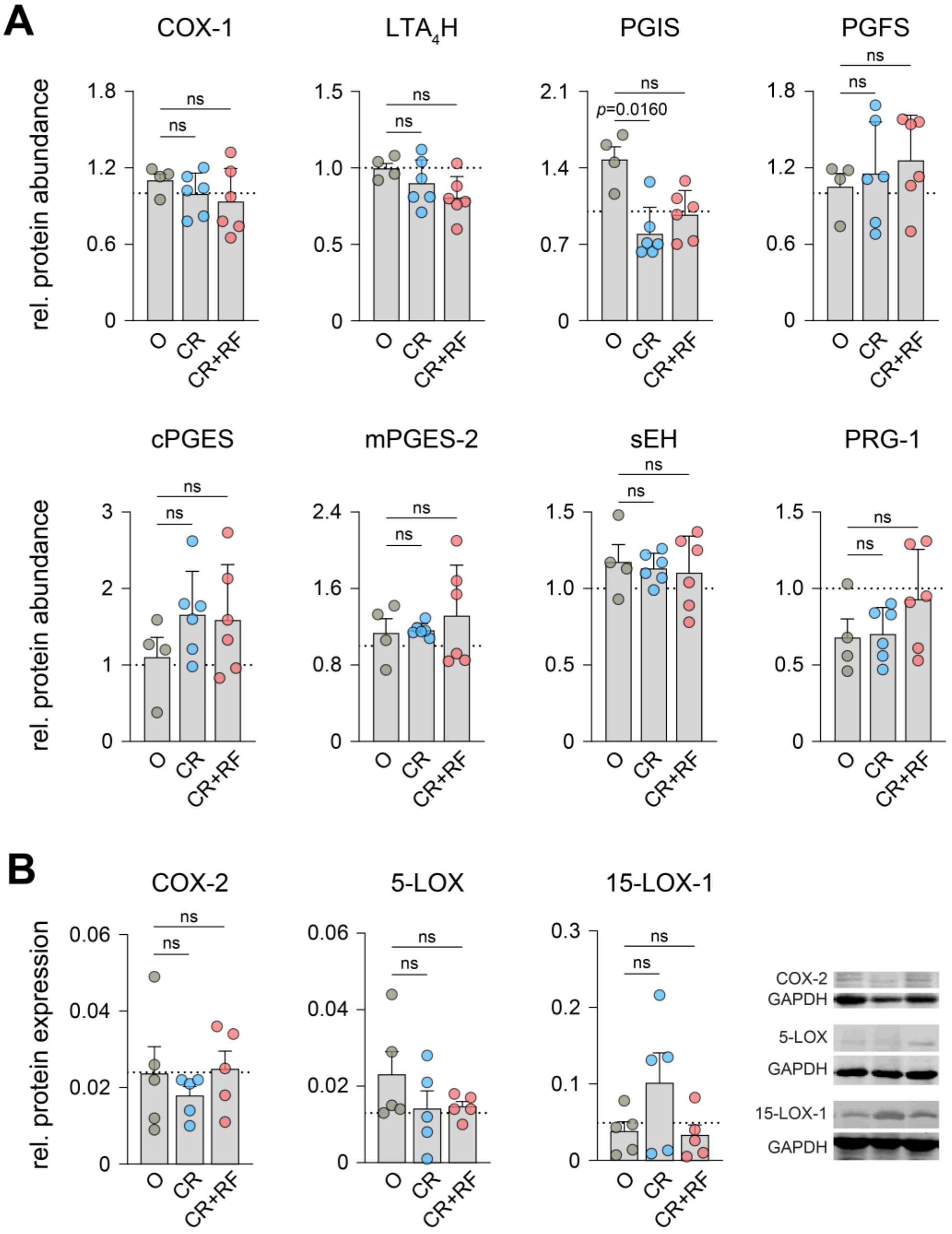

Figure S4: Influence of caloric restriction and re-feeding on LM biosynthetic enzymes.

(A) Protein levels of cyclooxygenase (COX-)1, leukotriene A_4 hydrolase (LTA_4H), prostaglandin (PG) I and F synthase, cytosolic PGE_2 synthase (cPGES), microsomal PGE_2 synthase (mPGES-)2, soluble epoxide hydrolase (sEH) and PG reductase (PRG)-1 in the hepatic proteome of young and old mice. Dotted line represents the mean value in young mice (Table S1, O: *n*=4; CR, CR+RF: *n*=6). **(B)** Relative expression of COX-2, 5-lipoxygenase (LOX) and 15-LOX-1 in the liver of old (O), CR and CR+RF was determined by SDS-PAGE and Western Blot (*n*=5). Dotted line represents the mean value in young mice (Fig. 2G).

Statistics: Data are shown as **(A, B)** mean ± SEM; *p*-values were calculated by one-way ANOVA for multiple comparisons with Tukey's post-hoc test or Brown-Forsythe and Welch ANOVA with Dunnett's T3 post-hoc test (Table S3); ns = not significant.

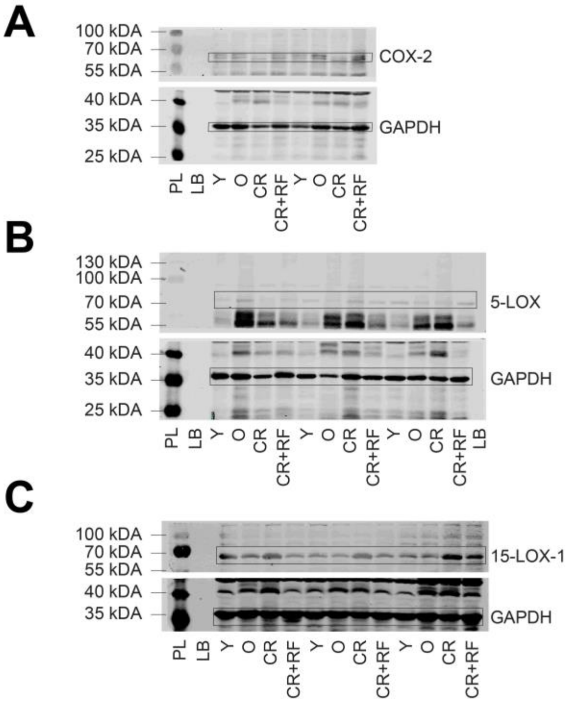

Figure S5: Raw images of Western blots of hepatic cyclooxygenase and lipoxygenases. Representative full scans of Western blots of **(A)** cyclooxygenase (COX)-2, **(B)** 5-lipoxygenase (LOX) and **(C)** 15-LOX-1 in whole liver lysates of young (Y), old (O), caloric restricted (CR), and re-fed (CR+RF) mice. Molecular weight markers are indicated on the left lanes. Lanes containing protein ladder (PL) or loading buffer (LB) are marked. The bands used for quantification (Fig. 2G, Fig. S2C) are highlighted by boxes.

Supplementary reference

1. Niu, L.; Thiele, M.; Geyer, P.E.; Rasmussen, D.N.; Webel, H.E.; Santos, A.; Gupta, R.; Meier, F.; Strauss, M.; Kjaergaard, M., et al. Noninvasive proteomic biomarkers for alcohol-related liver disease. *Nature Medicine* **2022**, *28*, 1277-1287, doi:10.1038/s41591-022-01850-y.
